# Human Platelets Utilize Cycloxygenase-1 to Generate Dioxolane A_3_, a Neutrophil-activating Eicosanoid*[Fn FN2]

**DOI:** 10.1074/jbc.M115.700609

**Published:** 2016-04-22

**Authors:** Christine Hinz, Maceler Aldrovandi, Charis Uhlson, Lawrence J. Marnett, Hilary J. Longhurst, Timothy D. Warner, Saydul Alam, David A. Slatter, Sarah N. Lauder, Keith Allen-Redpath, Peter W. Collins, Robert C. Murphy, Christopher P. Thomas, Valerie B. O'Donnell

**Affiliations:** From the ‡ Systems Immunity Research Institute and Division of Infection and Immunity, School of Medicine, Cardiff University, Cardiff CF14 4XN, United Kingdom,; the § Department of Pharmacology, University of Colorado at Denver, Aurora, Colorado 80045,; the ¶ Vanderbilt Institute of Chemical Biology, Centre in Molecular Toxicology, Vanderbilt-Ingram Cancer Center, Nashville, Tennessee 37232-0146, and; the ‖ William Harvey Research Institute, Queen Mary University of London, Charterhouse Square, London EC1M 6BQ, United Kingdom

**Keywords:** cyclooxygenase (COX), eicosanoid, eicosanoid biosynthesis, lipid, platelet, lipidomics

## Abstract

Eicosanoids are important mediators of fever, pain, and inflammation that modulate cell signaling during acute and chronic disease. We show by using lipidomics that thrombin-activated human platelets generate a new type of eicosanoid that both stimulates and primes human neutrophil integrin (Mac-1) expression, in response to formylmethionylleucylphenylalanine. Detailed characterization proposes a dioxolane structure, 8-hydroxy-9,11-dioxolane eicosatetraenoic acid (dioxolane A_3_, DXA_3_). The lipid is generated in nanogram amounts by platelets from endogenous arachidonate during physiological activation, with inhibition by aspirin *in vitro* or *in vivo*, implicating cyclooxygenase-1 (COX). Pharmacological and genetic studies on human/murine platelets revealed that DXA_3_ formation requires protease-activated receptors 1 and 4, cytosolic phospholipase A_2_ (cPLA_2_), Src tyrosine kinases, p38 MAPK, phospholipase C, and intracellular calcium. From data generated by purified COX isoforms and chemical oxidation, we propose that DXA_3_ is generated by release of an intermediate from the active site followed by oxygenation at C8. In summary, a new neutrophil-activating platelet-derived lipid generated by COX-1 is presented that can activate or prime human neutrophils, suggesting a role in innate immunity and acute inflammation.

## Introduction

Emerging evidence indicates that platelets influence innate immunity during acute infection and injury through their interactions with leukocytes (1–3). Platelets generate soluble lipid-signaling mediators that include eicosanoids such as thromboxane A_2_ (TXA_2_)^4^ and 12-hydroxyeicosatetraenoic acid (12-HETE) and small amounts of the prostaglandins (PGs) PGE_2_ and D_2_. Currently the effects of platelet-derived lipids on leukocytes are not fully known. In this study, we sought to discover whether platelets release leukocyte-regulating lipids using a lipidomic approach. Analogous methodologies have recently been used for discovery of lipids in diabetes, cardiovascular disease, and hemostasis (4–6).

Herein, we show that thrombin-activated human platelets generate a novel eicosanoid from endogenous substrate, proposed to be a dioxolane (DX), that elevates Mac-1 (CD11b/CD18) on neutrophils at nanomolar concentrations. We also present the detailed cellular and enzymatic mechanisms of formation along with characterization of its proposed covalent structure and established a quantitative assay. These data demonstrate a new platelet-derived leukocyte-activating eicosanoid and the first DX lipid to originate from mammalian cells, suggesting a novel mechanism for promoting neutrophil activities in the early stage of tissue damage/wounding responses.

## Experimental Procedures

### Materials

Lipids and lipid standards were purchased from Avanti Polar Lipids (Alabaster, AL) or Cayman Chemical (Ann Arbor, MI). Deuterated standards are as follows: arachidonic acid-*d*_8_, 5Z,8Z,11Z,14Z-eicosatetraenoic-5,6,8,6:54 PM 5/12/20169,11,12,14,15-*d*_8_ acid, ≥99% deuterated forms; PGE_2_-*d*_4_, 9-oxo-11α,15S-dihydroxy-prosta-5Z,13E-dien-1-oic-3,3,4,4-*d*_4_ acid, ≥99% deuterated forms; and PGD_2_-*d*_4_, 9α,15S-dihydroxy-11-oxo-prosta-5Z,13E-dien-1-oic-3,3,4,4-*d*_4_ acid, ≥99% deuterated forms. HPLC grade solvents were from Thermo Fisher Scientific (Hemel Hempstead, Hertfordshire, UK). PAR-1 and PAR-4 agonists were from Tocris Biosciences (Bristol, UK). COX-1 inhibitor (Sc-560) was from Cayman Chemical. Platelet signaling inhibitors (PP2, oleyloxyethylphosphocholine (OOEPC), bromoenol lactone, cytosolic phospholipase A_2α_ (cPLA_2α_) inhibitor (*N*-{(2*S*,4*R*)-4-(biphenyl-2-yl-methyl-isobutyl-amino)-1-[2-(2,4-difluorobenzoyl)-benzoyl]-pyrrolidin-2-ylmethyl}-3-[4-(2,4-dioxothiazolidin-5-ylidenemethyl)-phenyl]acrylamide, HCl), U73112, wortmannin, and p38 mitogen-activated protein kinase (MAPK) inhibitor were from Calbiochem (United Kingdom). Anti-human CD11b-Alexa Fluor 647 was from eBioscience. All other reagents were from Sigma unless otherwise stated. [^14^C]Arachidonic acid was from PerkinElmer Life Sciences; ovine COX-1 was from Cayman Chemical or purified as described (7, 8). Recombinant COX-2 was generated as described (9). *N-*Methyl benzohydroxamic acid (NMBHA) and 2,2′-azobis(4-methoxy-2,4,dimethyl valeronitrile) were kind gifts from Ned Porter (Vanderbilt University).

### Isolation of Human and Murine Platelets

Human blood donations were approved by the Cardiff University School of Medicine Ethics Committee, were with informed consent (SMREC 12/37 and SMREC 12/10), and according to the Declaration of Helsinki. For cPLA_2_-deficient samples, samples were approved by St Thomas's Hospital Research Ethics Committee, reference 07/Q0702/24: patient samples; South East NHS Research Ethics Committee. For studies on isolated platelets, whole blood was collected from healthy volunteers free from non-steroidal anti-inflammatory drugs for at least 14 days and added to acid/citrate/dextrose (ACD; 85 mmol/liter trisodium citrate, 65 mmol/liter citric acid, 100 mmol/liter glucose) (blood/ACD, 8.1:1.9, v/v) then centrifuged at 250 × *g* for 10 min at room temperature. Platelet-rich plasma was collected and centrifuged at 900 × *g* for 10 min, and the pellet was resuspended in Tyrode's buffer (134 mmol/liter NaCl, 12 mmol/liter NaHCO_3_, 2.9 mmol/liter KCl, 0.34 mmol/liter Na_2_HPO_4_, 1.0 mmol/liter MgCl_2_,10 mmol/liter Hepes, 5 mmol/liter glucose, pH 7.4) containing ACD (9:1, v/v). Platelets were centrifuged at 800 × *g* for 10 min and then resuspended in Tyrode's buffer at 2 × 10^8^·ml^−1^. Platelets were activated at 37 °C in the presence of 1 mmol/liter CaCl_2_ for varying times, with 0.2 units·ml^−1^ thrombin, 10 μg/ml collagen, 10 μmol/liter A23187, 20 μmol/liter TFLLR-NH_2_, or 150 μmol/liter AY-NH_2_ before lipid extraction as below. Experiments involving signaling inhibitors (1 mmol/liter aspirin, 1 μmol/liter SC-560, 10 μmol/liter indomethacin, 2 μmol/liter oleyloxyethylphosphocholine, 50 nmol/liter bromoenol lactone, 50 nmol/liter cPLA_2α_i, 75 μm thimerosal, 1 mm EGTA, 10 μm 1,2-bis(2-aminophenoxy)ethane-*N*,*N*,*N*′,*N*′-tetraacetic acid tetrakis(acetoxymethyl ester), 100 nm wortmannin, 100 nmol/liter Gö 6850, 50 μmol/liter PP2, 100 nmol/liter p38 MAPK inhibitor, 50 μm picotamide, 1–10 mm iodoacetate, and 5 μm U-73122) included a 10-min preincubation at room temperature. In some experiments, calcium was omitted from buffers. For separation of cells from microparticles, platelets were centrifuged at 970 × *g* for 5 min, and the supernatants were re-spun at 16,060 × *g* for 5 min. For aspirin supplementation, blood samples were first obtained following a 14-day nonsteroidal anti-inflammatory drug-free period for baseline determinations of eicosanoids. Subjects were administered 75 mg/day aspirin for 7 days, and they then provided a second blood sample. Platelets were isolated and activated *in vitro* using 0.2 unit/ml thrombin, as described above, and then lipids were extracted as described below. Exclusion criteria was a known sensitivity to aspirin. For studies on isolated murine platelets, whole blood was collected using cardiac puncture (mice were 28 weeks old) into 150 μl of ACD (85 mmol/liter trisodium citrate, 71 mmol/liter citric acid, 100 mmol/liter glucose). 150 μl of 3.8% sodium citrate and 300 μl of Tyrode's buffer (145 mmol/liter NaCl, 12 mmol/liter NaHCO_3_, 2.95 mmol/liter KCl, 1.0 mmol/liter MgCl_2_,10 mmol/liter Hepes, 5 mmol/liter glucose, pH 7.35) were added, and the blood was centrifuged at 150 × *g* for 5 min at room temperature. Platelet-rich plasma was collected, and 400 μl of Tyrode's buffer was added to the red cells and centrifuged again at 150 × *g* for 5 min at room temperature. Platelet-rich plasma was combined and centrifuged at 530 × *g* for 5 min at room temperature. Platelets were resuspended in Tyrode's buffer at 2 × 10^8^ ml^−1^. All animal experiments were performed in accordance with the 1986 United Kingdom Home Office Animals Act (Scientific Procedures). 12/15-LOX knock-out mice were generated as described previously (10), and wild-type male C57BL/6 mice (25–30 g) from Charles River, UK, were kept in constant temperature cages (20–22 °C) and given free access to water and standard chow.

### Isolation and Activation of Human Neutrophils

Human neutrophils were isolated from 20 ml of citrate anticoagulated whole blood and resuspended in Krebs buffer. Briefly, blood was mixed 1:3 with 2% trisodium citrate (w/v) and HetaSep (Stemcell Technologies) and allowed to sediment for 45 min at 20 °C. The upper plasma layer was recovered and underlaid with ice-cold Lymphoprep^TM^ (2:1 for plasma/Lymphoprep^TM^) and centrifuged at 800 × *g* for 20 min at 4 °C. The pellet was resuspended in ice-cold PBS and 0.4% sodium tricitrate (w/v) and centrifuged at 400 × *g* for 5 min at 4 °C. Contaminating erythrocytes were removed using up to three cycles of hypotonic lysis. Finally, cells were resuspended in a small volume of Krebs buffer (100 mmol/liter NaCl, 50 mmol/liter Hepes, 5 mmol/liter KCl, 1 mmol/liter MgCl_2_, 1 mmol/liter NaH_2_PO_4_, 1 mmol/liter CaCl_2_, and 2 mmol/liter d-glucose, pH 7.4), counted, and kept on ice. Neutrophils were diluted to 2 × 10^6^ cells/ml and incubated with or without DXA_3_ for 10 min at 37 °C. In some experiments, 10 μm fMLP was then added, and neutrophils were incubated for a further 10 min at 37 °C. Cells were blocked using 5% mouse serum in PBS (containing 0.5% BSA, 5 mmol/liter EDTA, and 2 mmol/liter sodium azide) for 1 h on ice and centrifuged at 320 × *g* for 5 min at 4 °C. Anti-human CD11b-Alexa Fluor 647 (0.0625 μg, eBioscience) or isotype control were added and incubated for 30 min on ice. Neutrophils were washed twice with ice-cold PBS (containing 0.5% BSA, 5 mmol/liter EDTA, and 2 mmol/liter sodium azide) and dissolved in the same buffer for flow cytometric analysis. Neutrophils were analyzed on a cyan ADP flow cytometer (Beckman Instruments) and identified by forward and side scatter and Alexa Fluor 647. DXA_3_ used for these experiments was purified from COX-1 incubations. Other lipids were not detectable in these preparations using MS.

### Culturing and Activation of RAW 264 Cells

RAW 264 cells were cultured in DMEM (10% FBS, 1× penicillin/streptomycin) at 37 °C and 5% CO_2_.

To determine PG synthesis, cells were incubated in serum-free DMEM (with 1× penicillin/streptomycin), and the cells were incubated for 1 h at 37 °C, 5% CO_2_. Where used, 200 ng/ml LPS was added, and cells were incubated for 24 h. Cells (8 × 10^6^ ml^−1^) were treated with 10 μm ionophore at 37 °C for 10 min, and lipids were extracted and analyzed as described below.

### Isolation of Human Serum

Whole blood from healthy volunteers was clotted at 37 °C for 15 min in glass and centrifuged (1500 rpm, 10 min, 4 °C). Serum was re-spun (2900 rpm, 10 min, 4 °C), and 3 volumes of MeOH/water (20:80 v/v) were added. Protein precipitates were spun down (13,000 rpm, 10 min, 4 °C), and supernatants were applied to preconditioned Waters C18 Sep-Pak columns. These were washed with 10 ml of water, 6 ml of hexane, and the eicosanoids were then eluted using 7 ml of methyl formate into tubes containing 6 μl of MeOH/glycerol (70:30 v/v) (11). Lipid were redissolved in methanol, chilled (−80 °C, 60 min), and re-spun (13,000 rpm, 10 min, 4 °C) before LC/MS/MS analysis for DXA_3_.

### Lipid Extraction

Lipids were extracted by adding a solvent mixture (1 mol/liter acetic acid, isopropyl alcohol, hexane (2:20:30, v/v/v)) to the sample at a ratio of 2.5 ml to 1 ml of sample, vortexing, and then adding 2.5 ml of hexane (12). Where quantitation was required, 5–10 ng of PGE_2_-*d*_4_, PGD_2_-*d*_4_, and 12-HETE-*d*_8_ were added to the samples before extraction, as internal standards. After vortexing and centrifugation, lipids were recovered in the upper hexane layer. The samples were then re-extracted by addition of an equal volume of hexane. The combined hexane layers were dried and analyzed for DXA_3_ using LC/MS/MS as below.

### Generation of DXA_3_ through Oxidation of 11-HPETE or by Purified or Recombinant COX Isoforms

Arachidonic acid was oxidized using *N*-methylbenzhydroxamic acid (NMBHA) and 2,2′-azobis(4-methoxy-2,4-dimethylvaleronitrile) (MeOAMVN) as detailed below. To a 6.5 μm arachidonic acid solution in chlorobenzene, 3.5 eq of NMBHA and 0.1 eq of MeOAMVN were added, and the mixture was stirred at 37 °C for 5 h under O_2_. After drying under N_2_, the sample was dissolved in methanol and stored at −80 °C until purification. Isolation of positional isomers used a Spherisorb ODS2 column (5 μm, 150 × 4.6 mm; Waters) with a gradient of 50–90% solvent B (acetonitrile, 0.1% formic acid) in solvent A (water, 0.1% formic acid) for 60 min, 90% solvent B for 4.5 min, and then re-equilibrating to 50% solvent B over 9.5 min with a flow rate 1 ml·min^−1^. Elution was monitored at 205 nm (unoxidized lipid) and 235 nm (HPETE). Fractions were collected and positional isomers identified using MS/MS transitions for the free HPETEs as follows: *m/z* 317.2 → 115.1 (5-HPETE); *m*/*z* 317.2 → 155.1 (8-HPETE); *m*/*z* 317.2 → 151.1 (9-HPETE); *m*/*z* 317.2 → 167.1 (11-HPETE); *m*/*z* 317.2 → 179.1 (12-HPETE); and *m*/*z* 317.2 → 219.1 (15-HPETE). Next, purified 11-HPETE was oxidized using 0.1 eq of MeOAMVN in 5 ml of chlorobenzene by stirring at 37 °C for 5 h under O_2_, and the hydroperoxides were then reduced to corresponding hydroxides using tin chloride (SnCl_2_). Lipids were extracted using the hexane/isopropyl alcohol extraction method, as described earlier.

Apo-COX-1 was stored in 80 mm Tris, pH 7.8, at −80 °C. In some experiments, a commercial preparation was used (Cayman Chemical). Wild-type murine COX-2 (recombinant) was at 10.61 mg·ml^−1^. For heme reconstitution, apo-COX-1 or -2 (35 μg) was preincubated on ice for 20 min with 2 molar equivalents of hematin in phosphate buffer (100 mm potassium phosphate buffer, pH 7.4). Then, 3.5 μg of the reconstituted enzyme was added to 1 ml of phosphate buffer and 500 μmol/liter phenol and incubated for 3 min at 37 °C in the presence of 150 μm arachidonate (AA or AA-*d*_8_). Where [^14^C]AA was used, 9.8 μg of enzyme was incubated with 70 μm AA (259 kBq). In some experiments, wild-type COX-2 was compared with active site mutants (V349A and W387F). The reaction was stopped by using ice-cold lipid extraction solvent and immediate extraction of lipids after addition of 5 ng each of PGE_2_-*d*_4_ and PGD_2_-*d*_4_ as internal standards, when required. In some experiments, 10 μm diethylenetriaminepentaacetic acid was added just before holo-COX-1. DXA_3_ was analyzed using reverse phase LC/MS/MS as described below.

In some experiments, AA was replaced with 1-stearoyl-2-arachidonyl-phosphatidylethanolamine. Free and esterified DXAs_3_ were analyzed using reverse phase LC/MS/MS as below.

### Reversed Phase LC/MS/MS and LC/MS^3^ of DXA_3_ and Platelet Eicosanoids

Several different LC separations were used on a 4000 Q-Trap platform. For high resolution mass analysis and fragmentation of free DXA_3_, a reversed-phase UPLC Fourier Transform MS method was used (Thermo Scientific Orbitrap Elite) using a Spherisorb ODS2 column (5 μm, 150 × 4.6 mm; Waters) with a flow rate of 1 ml·min^−1^. Solvent B was increased from 20 to 42.5% over 50 min, then increased to 90% over 10.5 min, held for 4 min, and then returned to 20% over 1 min. Equilibration time between runs was 4.5 min. Analysis was performed using heated ESI in negative ion mode at sheath, auxiliary and sweep gas flows of 70, 20, and 0, and capillary and source heater temperatures at 300 and 350 °C, respectively. LC/MS of parent ions was monitored using accurate mass in Fourier MS mode. Negative MS/MS spectra were acquired using higher energy collision-induced dissociation. Data-dependent MS^3^ of *m/z* 351 was carried out in ion trap-MS mode on the LTQ ion trap.

For MS/MS of *m/z* 351 or *m/z* 359, collision-induced dissociation (CID) was used with a resolving power of 30,000 in negative FTMS mode. Data-dependent MS^3^ of *m/z* 351 or *m/z* 359 from DXA_3_-*d*_8_ was carried out in negative FTMS mode with a resolving power of 15,000.

### Generation of a Quantitative Assay for DXA_3_

[^14^C]AA was oxidized using COX-1 as described for unlabeled AA above. The amount of [^14^C]DXA_3_ was determined by comparison with a [^14^C]AA standard curve analyzed using LC separation with radiochemical detection (Berthold Technologies) using a Spherisorb ODS2 column (5 μm, 150 × 4.6 mm; Waters) with a gradient of 20–42.5% solvent B (acetonitrile, 0.1% formic acid) in solvent A (water, 0.1% formic acid) over 50 min, 42.5–90% solvent B from 50 to 60 min, 90% solvent B from 60 to 64.5 min, 90 to 20% from 64.5 to 65.5 min and 20% solvent B from 65.5 to 75 min with a flow rate of 1 ml·min^−1^, and fractions were collected at 30-s intervals for LC/MS/MS confirmation of [^14^C]DXA_3_. The same gradient was also used for LC/MS/MS detection of [^14^C]DXA_3_ (*m/z* 353.2 → 165.1).

### Purification and Derivatization of DXA_3_ and GC/MS Analysis

DXA_3_ was purified from lipid extracts of thrombin-activated platelets or COX-1 reactions using HPLC/UV on a Spherisorb ODS2 column (5 μm, 150 × 4.6 mm; Waters) with a gradient of 20–42.5% solvent B (acetonitrile, 0.1% formic acid) in solvent A (water, 0.1% formic acid) over 50 min, 42.5–90% solvent B from 50 to 60 min, 90% solvent B from 60 to 64.5 min, 90 to 20% from 64.5 to 65.5 min, and 20% solvent B from 65.5 to 75 min with a flow rate of 1 ml·min^−1^, and fractions collected at 30-s intervals. DXA_3_-containing fractions were identified using MS using *m/z* 351.2 → 165.1, and then H_2_O was removed using Sep-Pak C18 cartridge purification (Waters). DXA_3_ was stored in methanol at −80 °C, prior to derivatization and GC/MS analysis.

#### 

##### 2,3,4,5,6-Pentaflourobenzyl Bromide Derivatization of Carboxyls

Lipid was dried under N_2_, and 25 μl of 1% 2,3,4,5,6-pentaflourobenzyl bromide and 25 μl of *N,N-*diisopropylethylamine, both in acetonitrile, were added. The mixture was vortexed and incubated for 30 min at 20 °C. The sample was dried under N_2_.

##### Methyloxime Derivatization of Carbonyl Groups

Lipids were dried under N_2_ in a glass vial. In a second vial, 1 ml of 1 n NaOH was combined with a few grains of methyloxime. The tubes were connected with the dry lipid in the uppermost tube and solvent in the lower tube to separate and prevent solvation and incubated for 2 h at 60 °C.

##### Trimethylsilane Derivatization of Hydroxyl Groups

Lipid was dissolved in 50 μl of *N,O-*bis(trimethylsilyl)trifluoroacetamide and 50 μl of acetonitrile, vortexed, and incubated for 1 h at 60 °C. The lipid was dried under N_2_, and 2 ml of ethyl acetate and 1 ml of H_2_O were added. The sample was vortexed, and the ethyl acetate layer was recovered, dried, and then dissolved in 1:2 H_2_O/methanol for LC/MS analysis or isooctane acetonitrile for GC/MS. GC/MS was carried out on a DSQ Thermo Finnigan as follows: source temperature, 200 °C; reagent gas, methane; gas flow, 1.8 ml/min; negative polarity, full scan 50–600. Column was a Phenomenex 30 m ZB-1.

### Acid Hydrolysis of DXA_3_

Semi-purified DXA_3_ generated by COX-1 was solubilized in acetonitrile (1 ml) before addition of 1% acetic acid (4 ml). Samples were left at room temperature for 30 min before extraction using a C_18_ solid phase extraction cartridge.

### Tin(II) Chloride Reduction of DXA_3_

DXA_3_ generated via oxidation of 11-HPETE was reduced using 95 μg of SnCl_2_ in water for 10 min at room temperature. Lipids were re-extracted as above using hexane/isopropyl/acetic acid.

### Statistics

Data on platelets are representative of at least three separate donors, with samples run in triplicate for each experiment. Data are expressed as mean ± S.E. of three separate determinations. Statistical significance was assessed using an unpaired two-tailed Student's *t* test. Where the differences between more than two sets of data were analyzed, one-way ANOVA was used followed by Bonferroni multiple comparisons test, as indicated in the figure legends. *p* < 0.05 was considered statistically significant.

## Results

### 

#### 

##### Platelets Generate DXA_3_

**FIGURE 1.**
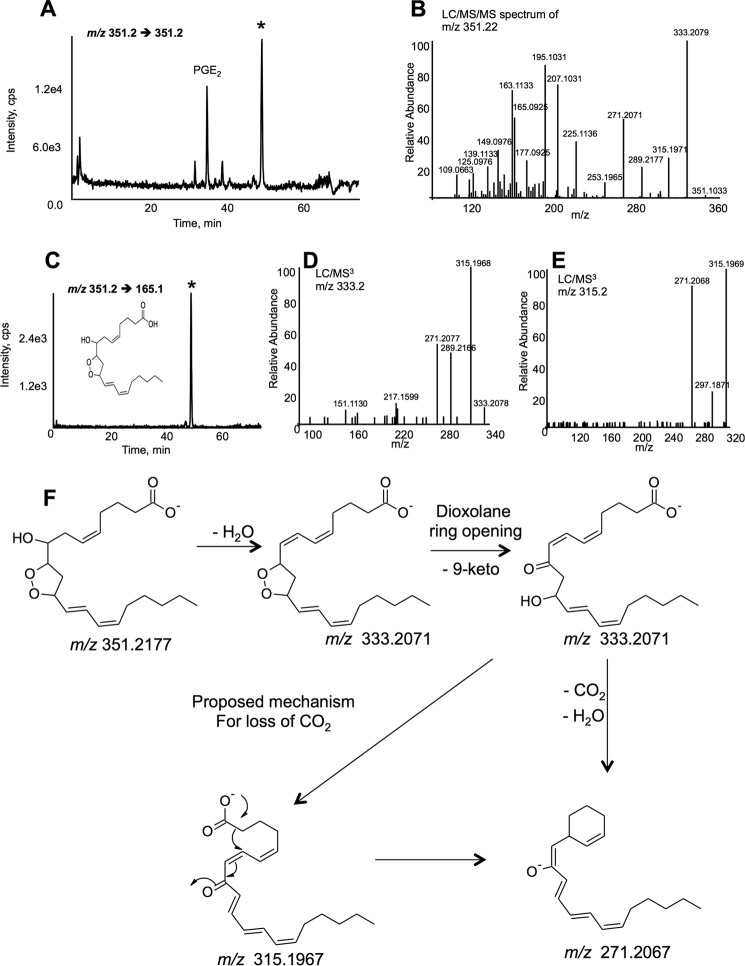
**DXA_3_ is generated by human platelets, characterization using MS/MS and MS^3^ fragmentation.** *A,* LC/MS/MS of DXA_3_ generated by thrombin-activated platelets. LC/MS/MS separation of lipids from thrombin-activated (0.2 units/ml, 30 min) platelets, using *m/z* 351.2 → 351.1, as described in the supplemental material, using a Q-Trap 4000. The later peak, labeled by (*), was identified as DXA_3_. *B,* MS/MS spectrum of DXA_3_. MS/MS of *m/z* 351.2 was acquired at the apex of the peak in *A* on an Orbitrap Elite. *C,* LC/MS/MS of DXA_3_. LC/MS/MS monitoring *m/z* 351.2 → 163.1 demonstrated a single peak for DXA_3_. *D,* LC/MS/MS of DXA_3_. Analysis was undertaken on the Orbitrap Elite in FTMS mode, separated by using reverse phase LC, isolated at *m/z* 351.2 in the Velos Pro, then fragmented by using CID at 50 V, with resolution 15,000 ppm, as described under “Experimental Procedures.” *E*, MS^3^ of daughter ion at *m*/*z* 333.2, with CID at 30 V. *C,* MS^3^ of daughter ion at *m*/*z* 315.2, with CID 30 V. *F,* proposed fragmentation pathway for *m*/*z* 351.2 generating *m*/*z* 333.2071, which fragments to *m*/*z* 271.2067 via *m*/*z* 315.1967. DXA_3_ loses H_2_O forming 333.2071. Following ring opening, leaving a keto group at C9, H_2_O, and CO_2_ are lost, generating *m/z* 271.2067 *via* a *m/z* 315.1967 intermediate, as shown.

We initially sought to discover esterified eicosanoids by scanning for precursors of *m/z* 351.2 in negative ion mode and of lipid extracts from thrombin-activated platelets. This work is published as the characterization of phospholipid esterified PGE_2_ and is described elsewhere (13). During precursor scanning for 351.2, we uncovered an unknown lipid also attached to phospholipids that was also generated as a free acid. This is visible when analyzing free acid lipids at *m/z* 351.2, where two lipids are seeded, including PGE^2^, and a more prominent ion at 48 min (*marked by* *, Fig. 1*A*) (13). MS/MS demonstrated a complex spectrum with major ions at *m/z* 163.2 and 165.2 that did not match any known eicosanoids in the LipidMaps database (Fig. 1*B*). However, a number of ions were indicative of prostaglandins, specifically *m/z* 333, 315, 289, and 271. The daughter ion at *m/z* 165.2 was then used to selectively detect the lipid in multiple reaction monitoring mode. A single lipid was visible at 48 min (Fig. 1*C*). The high resolution *m/z* [M − H]^−^ of 351.2177 suggests an elemental composition C_20_H_31_O_5_, corresponding to arachidonic acid plus three oxygen atoms, and the presence of 5 rings/double bonds (Fig. 1*B*). Its elution on reverse phase LC/MS/MS, considerably later than PGE_2_, indicated a less polar lipid (Fig. 1*A*).

**FIGURE 2.**
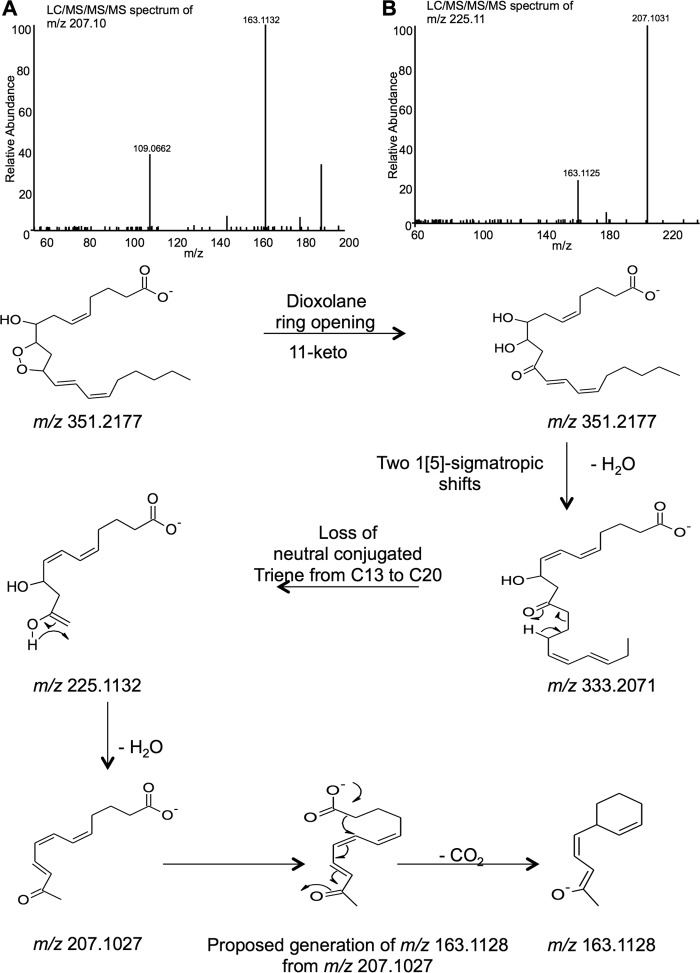
**Characterization of DXA_3_ MS/MS and MS^3^ fragmentation, using high resolution FTMS.** *A,* MS^3^ of daughter ion at *m*/*z* 207.1, with CID 30 V. *B,* MS^3^ of daughter ion at *m*/*z* 225.1, with CID 30 V. *Bottom panel,* proposed fragmentation pathway for *m*/*z* 351.2 generating *m*/*z* 333.2, then via fragmentation of 225.1, both at 207.1 and 163.1 are formed. Following ring opening, with keto group at C11, H_2_O is lost, followed by two 1[5]-sigmatropic shifts generating *m/z* 333.2071 Following loss of a conjugated triene, *m/z* 225.1132 is generated, which then loses H_2_O, and via an intermediate fragments to *m/z* 207.1027 and last 163.1128.

**FIGURE 3.**
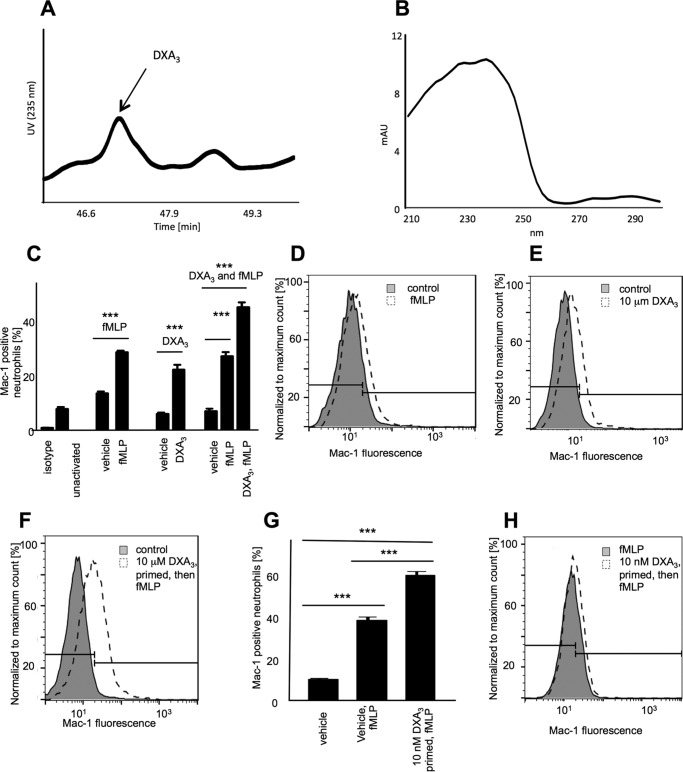
**DXA_3_ contains a UV chromophore and primes and activates neutrophil integrin expression.** *A,* DXA3 can be detected at 235 nm, during LC elution. DXA_3_ was generated using COX-1 as described under “Experimental Procedures” and then purified using LC/UV. *B,* DXA_3_ contains a UV chromophore. A UV spectrum was acquired at the apex of the peak at 46.7 and shows a λ_max_ at 238 nm. *C,* DXA_3_ activates neutrophil Mac-1 expression. Neutrophils were incubated with fMLP, DXA_3_, or both, before addition of anti-human CD11b (Mac-1)-Alexa Fluor 647 and flow cytometry analysis as under “Experimental Procedures.” A representative experiment repeated with three individual donors is shown (*n* = 3, mean ± S.E.). *D–F,* representative histograms depicting increased Mac-1 expression following activation with fMLP, DXA_3_, or fMLP/DXA_3_. *Line* represents the Mac-1-positive neutrophil gate, as set using untreated neutrophils. *G,* DXA_3_ primes neutrophil responses to fMLP. *Bar chart* showing activation of Mac-1 expression in three donors by fMLP with/without DXA_3_ priming for 10 min (*n* = 3 mean ± S.E.). *H,* representative histogram showing increased fMLP-stimulated Mac-1 expression following priming by DXA_3_. *Bar* represents the Mac-1 positive neutrophil gate, as set using untreated neutrophils. Statistical significance used Mann-Whitney *U* test, ****, *p* < 0.0001; ***, *p* < 0.001; **, *p* < 0.01. *n* = 3 donors.

Extensive structural characterization was undertaken using GC/MS, derivatization, MS/MS, and MS^3^ and suggested the lipid as DXA_3_. GC/MS presented in the supplemental material and demonstrates one hydroxyl and no carbonyls (supplemental Fig. 1). For further structural confirmation, DXA_3_ or DXA_3_-*d*_8_ generated by COX-1 was analyzed using high resolution MS*^n^* on an Orbitrap Elite, during LC elution. MS^3^ of daughter ions at *m/z* 333.1 and 315.2 indicate the origin of *m/z* 271.2 (Fig. 1, *D–F*), whereas MS^3^ of *m/z* 225.1 and 207.1 shows the origin of *m/z* 163.1 (Fig. 2). Of note, *m/z* 155 is a prominent ion generated on CID fragmentation of 8-HETE, thus supporting the position of the –OH group at C8. To further confirm, analogous experiments were undertaken using deuterated AA as substrate for DXA_3_ generation. MS^3^ of DXA_3_-*d*_8_ showed the same fragmentation pattern; however, many ions showed an additional ion at 1 atomic mass unit lower (*e.g. m/z* 340 and 321, as well as the expected 341 and 322), supporting our proposed fragmentation mechanisms as shown (supplemental Figs. 2 and 3). The lower *m/z* ions represent ring opening with addition of –H and concomitant loss of a single deuterium, as shown. Finally, a UV spectrum was acquired during purification of low nanogram amounts of COX-1-derived DXA_3_, showing a λ_max_ at 238 nm, similar to that reported for a similar DX by Teder *et al.* (14). This confirms the presence of a UV chromophore and is consistent with a conjugated diene at C12–15 of the lipid backbone (Fig. 3, *A* and *B*).

DXA_3_ is named based on the dioxolane structure, “A” for the first member of the class discovered, and 3 for the number of double bonds, as per traditional eicosanoid naming conventions (15). Further work is required to confirm the structure using NMR and to determine enantiomeric/geometric isomer composition, using synthetic standards once these become available.

##### DXA_3_ Activates Neutrophil Surface Integrin Expression

Neutrophils incubated with purified DXA_3_ elevated surface Mac-1 (integrin, CD11b/CD18), comparable with fMLP activation (Fig. 3, *C* and *D*). Together, fMLP and DXA_3_ caused an additive effect on Mac-1 expression, suggesting they activate neutrophils by distinct pathways (Fig. 3, *C–F*). However, at 10 nm, DXA_3_ effectively primed for fMLP activation after a 10 min pre-incubation (Fig. 3, *G* and *H*).

##### Human Platelets Acutely Generate DXA_3_ on Thrombin Activation via COX-1

**FIGURE 4.**
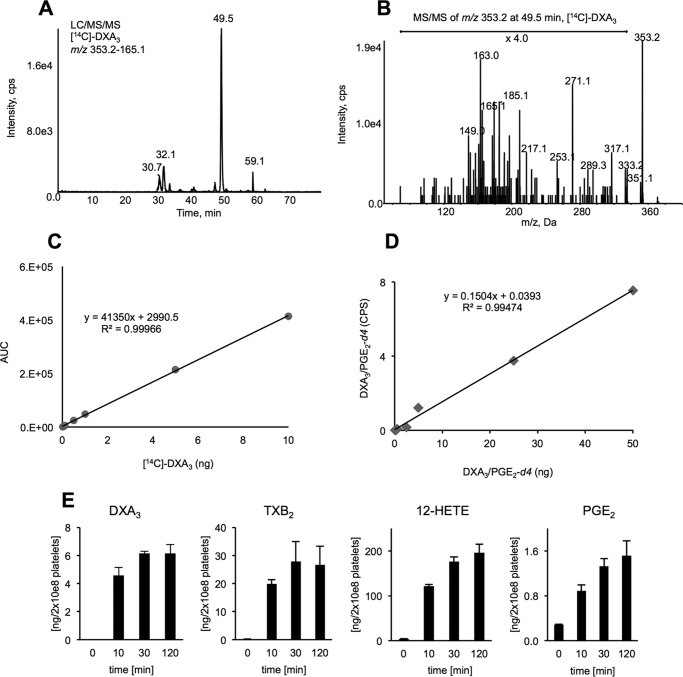
**Setting up a quantitative assay for DXA_3_ and determining its levels in human platelets.** [^14^C]DXA_3_ was generated, using COX-1 and purified using HPLC with radiochemical detection, as described in the supplemental material. [^14^C]DXA_3_ was quantified by comparing the radiochemical response to [^14^C]AA. *A,* LC/MS/MS analysis of *m/z* 353.2 → 165.1 showing [^14^C]DXA_3_ eluting at 49.51 min, undertaken on the 4000 Q-Trap as described under “Experimental Procedures.” *B,* MS/MS spectrum of [^14^C]DXA_3_ showing *m*/*z* 353.2 as parent ion. Experiment was performed on the 4000 Q-Trap platform in enhanced product ion mode, as described under “Experimental Procedures.” *C,* standard curve for [^14^C]DXA_3_ using LC/MS/MS detection used for quantification of unlabeled DXA_3_. *D,* standard curve for quantitation of DXA_3_ in biological samples. A standard curve was generated with varying DXA_3_ but keeping PGE_2_-*d**_4_*** levels constant, and responses were plotted as shown. *E,* time course of eicosanoid generation by thrombin-activated platelets. Washed platelets were activated for varying times, using 0.2 unit·ml^−1^ thrombin, then lipids were extracted and analyzed using reverse phase LC/MS/MS, monitoring parent *m/z* 351.2 → 165.1 (DXA_3_), 319.2 → 179.1 (12-HETE), *m/z* 351.2 → 271.1 (PGE_2_), and *m/z* 369.2 →169.1 (TXB_2_), as described under “Experimental Procedures.” Data are representative of experiments repeated three times on different donors (*n* = 3, mean ± S.E.). *F,* levels of eicosanoids generated by genetically unrelated volunteers. Data are shown as Tukey *boxplots*, where *whiskers* represent 1.5 the lower and upper interquartile range, data not included within the whiskers are displayed as an outlier. Statistical significance used Mann-Whitney *U* test, ****, *p* < 0.0001; ***, *p* < 0.001; **, *p* < 0.01. *n* = 7–10 donors.

As a purified standard is not yet available, we synthesized and purified a biogenic standard by COX-1 oxidation of [^14^C]AA oxidation *in vitro*, using COX-1. This was quantified using radiochemical detection and HPLC-purified. The radiolabeled standard was then utilized in LC/MS/MS assays, to quantify cold DXA_3_ generated by COX-1, which was then used as a primary standard for quantitation, against PGE_2_-*d*_4_ as internal standard. Fig. 4, *A–D,* shows the LC/MS/MS and MS/MS spectrum of purified [^14^C]DXA_3_ along with standard curves for both radiochemical detection of [^14^C]DXA_3_ and DXA_3_ *versus* PGE2-*d*_4_.

**FIGURE 5.**
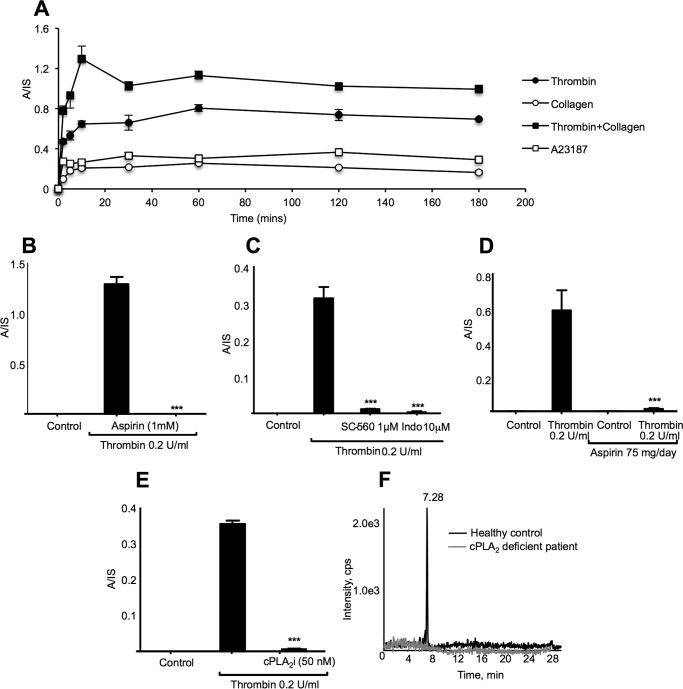
**Time course of agonist-stimulated generation and demonstrating the requirement for COX-1 and cPLA_2_ in DXA_3_ formation.** *A,* generation of DXA_3_ by human platelets. Washed platelets were activated for varying times, using 0.2 unit·ml^−1^ thrombin, 10 μg/ml collagen, 10 μmol/literA23187, and then lipids were extracted and analyzed using reverse phase LC/MS/MS, monitoring parent *m/z* 351.2 → 165.1 as described under “Experimental Procedures.” Levels are expressed as analyte/internal standard. Data are representative of experiments repeated at least three times on different donors (*n* = 3, mean ± S.E.). *B* and *C,* requirement for COX-1 for DXA_3_ formation. Platelets were incubated with inhibitors 10 min prior to thrombin activation (0.2 units/ml for 30 min at 37 °C). Lipids were extracted and analyzed using LC/MS/MS monitoring *m/z* 351.2 → 165.1, as described under “Experimental Procedures.” Data are representative of experiments repeated at least three times on different donors (*n* = 3, mean ± S.E.). ***, *p* < 0.001 *versus* thrombin, using ANOVA and Bonferroni post hoc test. Inhibitors used were aspirin, SC-560 (COX-1 selective), or indomethacin/aspirin (non-selective COX inhibition). *D, in vivo* aspirin blocks platelet DXA_3_ generation. Lipids were analyzed following thrombin activation of washed platelets, before or after supplementation with 75 mg/day aspirin for 7 days. Data are representative of five independent donors (*n* = 5, mean ± S.E.); ***, *p* < 0.001 *versus* thrombin alone, using ANOVA and Bonferroni post hoc test. *E,* cPLA_2_ is required for DXA_3_ formation. Platelets were incubated with 50 nm cPLA_2_ inhibitor (*cPLA_2_i*) 10 min prior to thrombin activation (0.2 units/ml for 30 min at 37 °C). Lipids were extracted and analyzed using LC/MS/MS monitoring *m/z* 351.2 → 165.1, as described under “Experimental Procedures.” Data are representative of experiments repeated at least three times on different donors (*n* = 3, mean ± S.E.). ***, *p* < 0.001 *versus* thrombin, using ANOVA and Bonferroni post hoc test. *F,* platelets genetically deficient in cPLA_2_ do not generate DXA_3_. Washed human platelets from a patient genetically deficient in cPLA_2_ or a healthy control were activated using thrombin (0.2 units/ml for 30 min at 37 °C) before lipid extraction and analysis using reverse phase LC/MS/MS, monitoring *m/z* 351.2 → 165.1 as described under “Experimental Procedures.”

**FIGURE 6.**
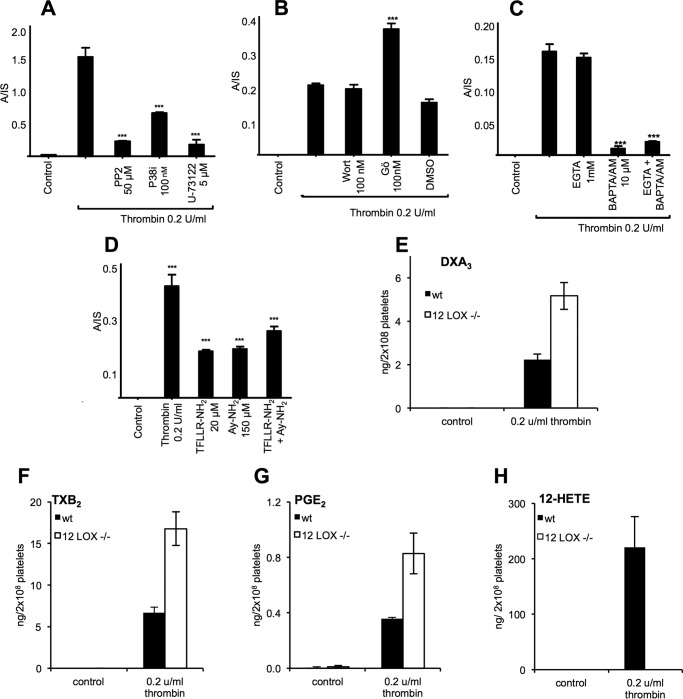
**Demonstration of a coordinated signaling pathway leading to DXA_3_ formation in thrombin-activated platelets, its elevated formation in murine platelets deficient in 12-LOX.** *A–C,* effects of signaling inhibitors on DXA_3_ formation. *A,* PP2, 50 μm (*src* family tyrosine kinase), p38 inhibitor, 100 nm (p38 MAPK), or U-73112, 5 μm PLC. *B,* wortmannin, 100 nm (PI 3-kinase), Gö 6850 (PKC), 100 nm (PKC), or vehicle (DMSO, 0.5%). *C,* EGTA 1 mm (extracellular Ca^2+^) or 1,2-bis(2-aminophenoxy)ethane-*N*,*N*,*N*′,*N*′-tetraacetic acid tetrakis(acetoxymethyl ester) 10 μm (intracellular Ca^2+^). *D,* DXA_3_ is generated via PAR-1 and PAR-4 receptor stimulation. Washed platelets were activated with a PAR-1 agonist, TFLLR-NH_2_ (20 μm), and/or a PAR-4 agonist, AY-NH_2_ (150 μm), for 30 min at 37 °C and then analyzed as described under “Experimental Procedures.” ***, *p* < 0.001 *versus* control, using ANOVA and Bonferroni post hoc test. *E,* generation of DXA_3_ by murine platelets is enhanced in 12-LOX deficiency. Murine platelets were activated using 0.2 units/ml thrombin for 30 min before lipids were extracted and analyzed using LC/MS/MS. *F–H,* generation of eicosanoids by murine platelets. Murine platelets were activated using 0.2 units/ml thrombin for 30 min before lipids were extracted and analyzed using LC/MS/MS.

**SCHEME 1.**
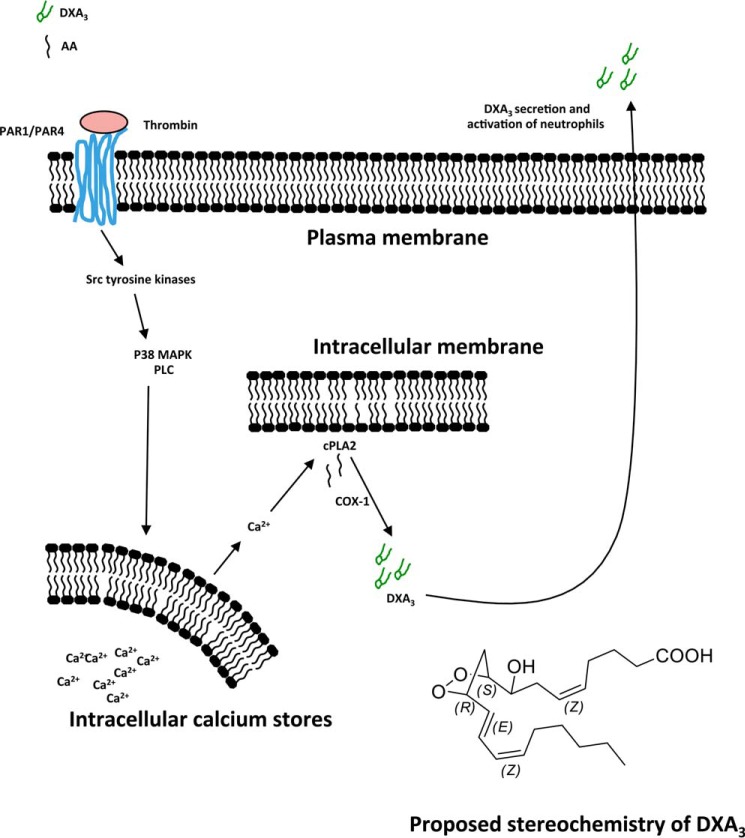
**Summary of cellular synthesis pathway for free DXA_3_ by human platelets.** Thrombin triggers platelet activation via PAR1 and PAR4 and then intracellular signaling via Src tyrosine kinases, MEK, MAPK, PLC, and intracellular Ca^2+^. Activation of cPLA_2_ leads to AA release, which is then oxidized via COX-1 forming DXA_3_. Free DXA_3_ is secreted to interact with neighboring cells, including neutrophils.

DXA_3_ was undetectable basally with levels rising by 10 min of thrombin activation (*n* = 7–10 separate donors, mean ± S.E.). Levels were higher than PGE_2_, but lower than TXB_2_ or 12-HETE (a representative donor is shown in Fig. 4*E*, data for all donors as Fig. 4*F*). Levels varied between genetically unrelated donors for all eicosanoids. DXA_3_ formed on activation by collagen, ionophore, or collagen/thrombin of platelets, with high levels already apparent 2 min post-activation (Fig. 5*A*). Its thrombin-dependent formation was blocked by the selective COX-1 inhibitor, SC560, aspirin, or indomethacin *in vitro* or *in vivo* following administration of 75 mg/day aspirin for 7 days in healthy donors (Fig. 5, *B–D*). DXA_3_ was absent in thrombin-activated platelets from a patient with genetic deficiency of cPLA_2_ (16) or in the presence of the cPLA_2_ inhibitor, cPLA2i (Fig. 5, *E* and *F*). In contrast, neither iPLA_2_ nor sPLA_2_ appeared significantly involved (data not shown). Pharmacological inhibitors/agonists implicated PAR-1 and -4 receptors, *src*-tyrosine kinase, p38 MAPK, intracellular calcium, and PLC, but ruled out phosphatidylinositol 3-kinase, although PKC played an inhibitory role (Fig. 6, *A–D*). Murine platelets also generated DXA_3_ at levels similar to human cells; however, levels were significantly higher in platelets genetically deficient in a second arachidonate-oxidizing enzyme, 12-lipoxygenase (LOX) (Fig. 6*E*). Similarly, other COX-1-derived lipids were elevated in these platelets, and 12-HETE-was absent (Fig. 6, *F–H*). Collectively, these data show that thrombin-stimulated DXA_3_ generation depends on a highly coordinated signaling pathway, culminating in cPLA_2_-dependent hydrolysis of AA from phospholipids, prior to its oxygenation by COX-1 (Scheme 1).

##### Elucidating the Mechanism of DXA_3_ Generation by COX Isoforms

**FIGURE 7.**
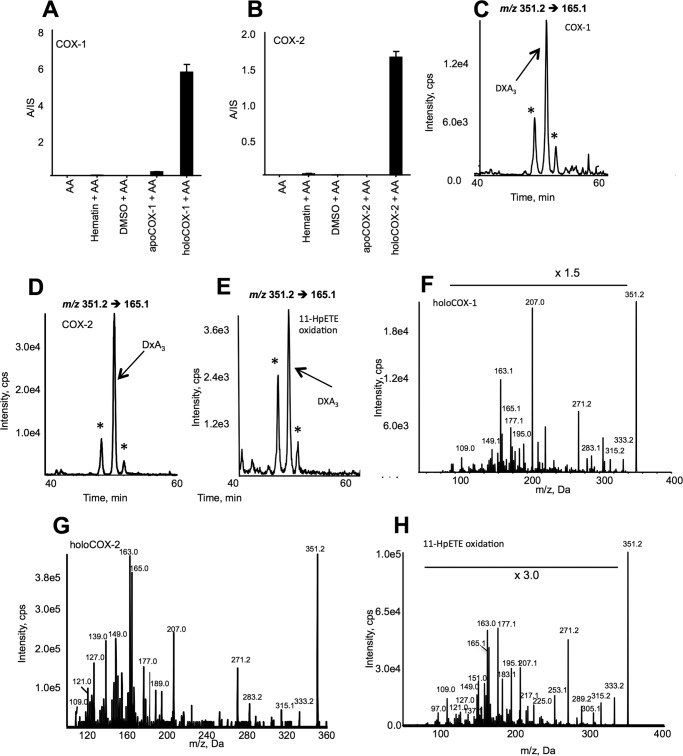
**DXA_3_ is generated by purified COXs and via oxidation of 11-HPETE.** *A* and *B,* COX isoforms generate DXA_3_. 3.5 μg of apo- or holo-COX-1 or COX-2, or hematin (control for reconstituted enzyme), or DMSO (vehicle for hematin) was incubated with 150 μm of AA for 3 min at 37 °C, before lipid extraction and analysis as described under “Experimental Procedures.” Levels are expressed as ratio analyte to internal standard/3.5 μg of enzyme generated over 3 min (*n* = 3, mean ± S.E.). Data are representative of ≥3 separate experiments. *C* and *D,* LC/MS/MS of DXA_3_ formed *in vitro* via COX-1 or -2. Lipid extracts were separated using reverse phase LC/MS/MS, monitoring *m/z* 351.2 → 165.1, with reactions as described under “Materials and Methods.” *E,* LC/MS/MS of DXA_3_ formed *in vitro* via 11-HPETE oxidation. Purified 11-HPETE was oxidized as described under “Experimental Procedures” and separated using LC/MS/MS. *F* and *G,* MS/MS spectra of DXA_3_ formed *in vitro* via COX-1 or -2. Lipid extracts were separated as in *C* and *D*. MS spectra were acquired at the apex of elution of DXA_3_. *H,* MS/MS spectra of DXA_3_ formed *in vitro* via 11-HPETE oxidation. Lipid extracts were separated as in *C* and *D*. MS spectra were acquired at the apex of elution of DXA_3_. * shows position of additional isomers with identical MS/MS spectra to DXA3 eluting either before or after lipid.

To test whether DXA_3_ could be formed by COX turnover, we examined the incubations of COX-1 or -2 with AA and also synthesized the free acid form of DXA_3_, through oxidation of 11-HPETE, as described by Porter and co-workers for generating cholesteryl-esterified dioxolane lipids (17, 18). In all reactions, an ion with same retention time and MS/MS spectrum as the platelet lipid was formed (Fig. 7, *A–H*). However, for either COX or 11-HPETE oxidation-generated DXA_3_, two additional ions with *m/z* 351.2 →165.1 were seen eluting just before and after DXA_3_, which may represent enantiomers or positional isomers, *e.g.* at C8, C9, or C11. Monocyclic isomers contain three chiral centers, thus eight possible stereoisomers or four pairs of enantiomers): RRR, RRS, RSS, SSS, SSR, SRR, SRS, and RSR (19). These additional ions show identical MS/MS spectra to DXA_3_ (data not shown) suggesting these peaks to be isomers. With enantiomers not being separated by our chromatography, up to four peaks of isomers would be expected. The absence of these isomers in platelet extracts (Fig. 1*C*) indicates a higher degree of control over the cellular biosynthesis of DXA_3_, preventing generation of stereoisomers. This may indicate that additional unknown enzymatic pathways exert control of DXA_3_ generation in platelets and will be subject to further study.

**FIGURE 8.**
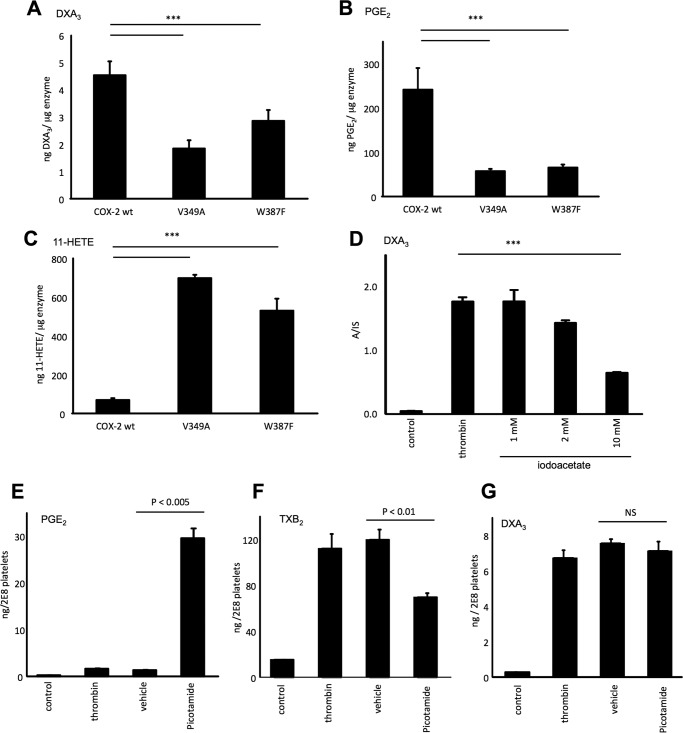
**DXA_3_ exits the COX active site downstream of 11-LOO^•^ radical escape, although peroxidase activity but not thromboxane synthase is involved in platelet DXA_3_ generation.** *A–C,* COX-2 mutants that generate more 11-HETE form less DXA_3_ during turnover. DXA_3_, PGE_2_, and 11-HETE generated by COX-2 wild type (*WT*) or mutants (V349A and W387F). Following reconstitution with hematin, 30 μm arachidonate was oxidized using 10.2 μg of enzyme at 37 °C for 5 min under O_2_ atmosphere. *n* = 5–7, ***, *p* < 0.05 (single factor ANOVA followed by two-tailed *t* test). *D,* peroxidase turnover is required for DXA_3_ generation. Platelets were treated with 1–10 mm iodoacetate before thrombin activation and analysis of DXA3 using LC/MS as described under “Experimental Procedures.” One representative donor, triplicates ± S.E., single factor ANOVA followed by Bonferroni ***, *p* < 0.005 are shown. *E–G,* thromboxane synthase is not involved in platelet DXA_3_ generation. Platelets were incubated with 50 μm picotamide 10 min prior to thrombin activation (0.2 units/ml for 60 min at 37 °C). Lipids were extracted and analyzed using LC/MS/MS monitoring *m/z* 351.2 → 165.1, as described under “Experimental Procedures.” Data are representative of experiments repeated at least three times on different donors (*n* = 5, mean ± S.E.). ***, *p* < 0.001 *versus* thrombin, using ANOVA and Bonferroni post hoc test. *NS*, not significant.

**SCHEME 2.**
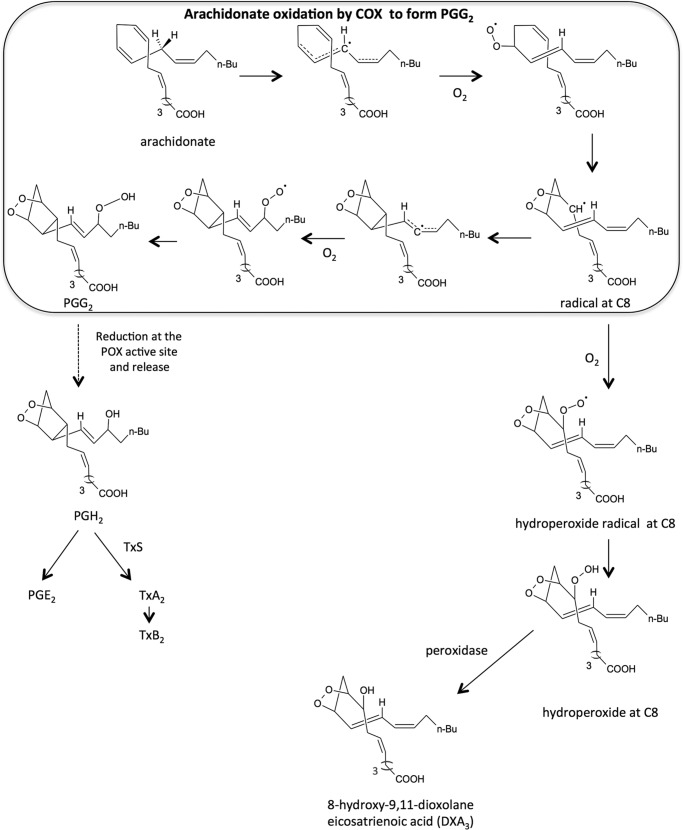
**Proposed mechanism of DXA_3_ formation by COX.** During COX turnover, a dioxolane ring forms between C9 and C11, prior to prostanoid ring formation, resulting in a carbon-centered radical at C8. Leakage of this lipid intermediate from the active site, then addition of oxygen followed by reduction to LOOH, and then LOH leads to formation of DXA_3_.

Platelets generate significant amounts of 11-HETE via COX-1 turnover (20, 21). This likely results from 11-hydroperoxyl radical intermediates (11-LOO^•^) exiting the catalytic site, then being reduced to form 11-HETE. Similarly, we reasoned that DXA_3_ could form by COX via rearrangement of an enzyme-generated intermediate exiting the active site early, before full prostanoid ring formation. This could occur either at the 11-LOO^•^ or 9,11-dioxolane radical stage (*e.g.* just before or after formation of the DX ring). To examine this, we measured DXA_3_ formation by two COX-2 mutants that generate more 11-HETE and less PGH_2_ than wild-type enzyme (22). Thus, these enzymes favor escape of lipid radicals prior to DX/prostanoid ring formation. Both mutants were found to generate less DXA_3_, indicating that the DX ring forms before DXA_3_ leaves the active site (Fig. 8, *A–C*, and Scheme 2). Following escape of a 9,11-DX radical, oxygen addition at C8 is expected, followed by peroxidase-dependent reduction. This could be mediated by COX-1 peroxidase or GSH peroxidase. In support, DXA_3_ formation was inhibited by 1–10 mm iodoacetate, a thiol-alkylating reagent (Fig. 8*D*).

##### Generation of DXA_3_ Is Independent of Thromboxane Synthase

To determine the role of enzymatic activities downstream of COX-1, an inhibitor of thromboxane synthase was added to platelets during activation. Picotamide led to inhibition of TXB_2_ generation and a corresponding elevation in PGE_2_, because less PGH_2_ was being converted by thromboxane synthase (Fig. 8, *E* and *F*). However, DXA_3_ formation was unaffected (Fig. 8*G*). Last, there was no correlation between TXB_2_ and DXA_3_ levels, further supporting the idea that thromboxane synthase is not involved in DXA_3_ generation (data not shown).

##### Generation of DXA_3_ by RAW 264 Cells and Human Serum

**FIGURE 9.**
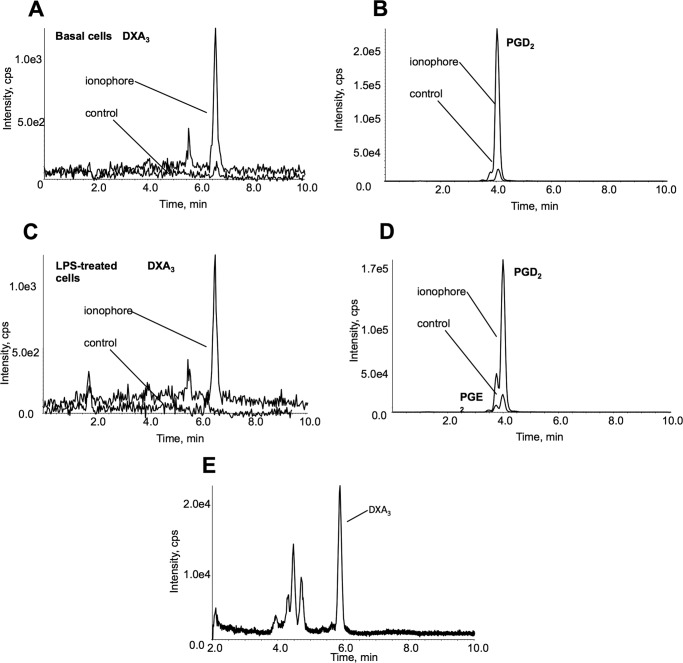
**DXA_3_ is generated by RAW cells and during physiological coagulation.** *A–D,* RAW cells generate DXA_3_ under basal non-inflammatory conditions. RAW cells were incubated in serum-free DMEM for 1 h at 37 °C, 5% CO_2_. Where used, 200 ng/ml LPS was added for 24 h. Cells (8 × 10^6^ ml^−1^) were activated using 10 μm A23187 at 37 °C for 10 min, and lipids were extracted and analyzed using LC/MS/MS. DXA_3_ was monitored using *m/z* 351.2 to 165.1 and PGE_2_/D_2_ using *m/z* 351.2 to 271.1 utilizing a 4000 QTrap. *A* and *B,* LC/MS/MS of basal RAW cells with/without 10 μm A23187. *C* and *D.* LC/MS/MS of LPS-treated RAW cells with/without 10 μm A23187. *E,* DXA_3_ is generated during physiological blood clotting. Whole blood was clotted, serum was harvested, and lipid was extracted as described under “Experimental Procedures.” LC/MS/MS was performed as for free DXA_3_ on a Q-Trap platform. Note that retention time of serum and RAW cell DXA3 differs slightly because these were analyzed several months apart on different columns. The identities have been confirmed through co-elution with platelet DXA3 (data not shown).

To determine generation of DXA_3_ in other cell types, RAW 264 macrophages were treated using LPS for 24 h, with/without ionophore activation. Under basal conditions, these cells express only COX-1, although following LPS treatment, they up-regulate COX-2. We found that cells required ionophore for robust PG generation. DXA_3_ formation paralleled that of PGD_2_, being present basally, but unaffected by inflammatory activation. Thus, the lipid was most likely generated by COX-1 but not COX-2 in these cells (Fig. 9, *A–D*). Human blood was harvested and allowed to clot. Analysis of serum demonstrated a significant DXA_3_ peak, indicating that physiological coagulation forms this lipid (Fig. 8*E*). In contrast, DXA_3_ was absent from plasma (data not shown).

## Discussion

Herein, we used a lipidomic approach to identify and characterize a new neutrophil-activating lipid, proposed to be DXA_3_, formed endogenously by agonist-activated platelets in a COX-1-dependent manner, by a macrophage cell line, and during blood clotting. At this time, we present a proposed structure based on strong and consistent UV, GC/MS, and LC/MS*^n^* data. Once the sufficient synthetic standard is available, full NMR analysis will be undertaken. We note that many other biologically relevant lipids, including thromboxane, leukotrienes, protectins, etc., were first published as proposed structures in a similar manner to our study.

The mechanism of DXA_3_ formation *in vitro* by COX enzymes is described, as well as its detailed cellular biosynthesis pathway in human platelets. DXA_3_ represents the first DX eicosanoid isolated and characterized within cells. To date, these have only been demonstrated to form via chemical oxidation of purified arachidonate esters or ω3 fatty acids or by *in vitro* lipoxygenase oxidation of epoxides, and neither their generation by cells nor any bioactivities have been described (14, 18, 19, 24, 25, 27, 28). Our study greatly extends these old *in vitro* observations by demonstrating that DX lipids are not only generated by live primary cells under physiological conditions, but they possess biological activity of relevance to innate immunity. This study places this eicosanoid in a new family of products likely relevant as a lipid mediator as are the prostaglandins, leukotrienes, and P450-derived eicosanoids. Extending these cell biology studies to *in vivo* measurements of leukocyte function and inflammation will be undertaken as soon as the synthetic standard becomes available.

Eicosanoids are essential lipid signaling mediators involved in diverse biological processes (29–32). Identification of new bioactive eicosanoids from this pathway could pave the way for additional and more selective therapeutic approaches. Thus, the proposed structure for DXA_3_ represents a new member of this family, characterized by a unique five-membered endoperoxide ring, and generated by a COX isoform known to play important roles in vascular disease and, more recently, in cancer.

Mac-1 (CD11b/CD18) is the predominant β2 integrin on neutrophils that mediates adhesion-dependent processes, such as binding to the endothelium or phagocytosis, recruitment, and transendothelial migration (33, 34). Herein, we show that DXA_3_ enhances Mac-1 on the cell surface (Fig. 3). The only other known Mac-1-inducing eicosanoids are leukotriene B4 and 5-oxo-ETE, both neutrophil-derived lipids (35, 36). Thus, neutrophil integrin activation by platelet-derived DXs could be of relevance during acute inflammation and infection. DXA_3_ was generated by platelets utilizing endogenous substrate in nanogram amounts that are ∼10-fold higher than platelet PGE_2_ (Fig. 4*E*). Its formation does not require supply of exogenous substrates and can be triggered directly by pathophysiological agonists in healthy primary cells, both important criteria in establishing that a new lipid mediator is endogenously relevant.

As DXA_3_ was generated via COX-1 in platelets, we reasoned that it could form through two potential mechanisms, either (i) rearrangement of 11-LOO^•^, known to be released by the enzyme during turnover, or (ii) that the dioxolane ring could form before the lipid exits from the active site (20, 21, 23, 37). In both cases, attack at C9 by the peroxyl radical would form the 9,11-dioxolane, which would be followed by oxygen addition at C8, and finally peroxidase reduction of the resulting LOOH by COX-1 peroxidase or GSH peroxidase in platelets. Our data using mutant COX-2 enzymes that generate less DXA_3_ but more 11-HETE suggest that the DX ring forms before lipid release by the enzyme. Thus, dioxolane ring formation occurs first and before prostanoid ring closure between C8 and C12 (Scheme 2). Finally, given that COX-1 generates 11R-HETE, we postulate that the dioxolane ring will likely be 9*S*,11*R*. Our observation of a single DX isomer in platelets but several in purified enzyme reactions indicates that platelets exert additional control over its biosynthesis. This may be at the stage of oxygen insertion into the chiral center at C8.

DXA_3_ was generated by platelets via a highly coordinated sequence of signaling events, including PAR-1 and -4, *src* tyrosine kinases, intracellular Ca^2+^, cPLA_2_, PLC, p38, and MAPK. This indicates tight control of its formation, similar to generation of other COX metabolites, such as TXA_2_. The signaling pathway is distinct from generation of free and esterified HETE and hydroxydocosahexadienoic acids, which form via 12-LOX, and require extracellular calcium, independent of PLC and MAPK (6, 26).

DXA_3_ was also generated by RAW cells as a single isomer, similar to platelets. Our preliminary data suggest that it originates primarily from COX-1 in these cells. In contrast, we found that either isoform could generate the lipid *in vitro*. In line with our observation that cellular DXA_3_ is a single isomer in platelets and RAW cells, although three isomers form via COXs *in vitro*, this collectively suggests that cellular DXA_3_ generation is under enzymatic control downstream of its synthesis by COX-1. Future studies will examine the ability of cellular COX-2 to generate the isoform and under which activation conditions. COX-1 is important not only in acute innate immunity but also in gastric function and development, and thus its generation by this isoform may have wider implications for eicosanoid biology in other organs.

Murine platelets also generated DXA_3_, and levels of this were enhanced in cells deficient in 12-lipoxygenase. This may be related to greater availability of substrate, although this has not been explored herein.

Eicosanoids include a large number of related structures formed via oxidation of arachidonate, following its release from intracellular membranes by phospholipases. A rapid burst of eicosanoid generation is a key event during cell activation and is stimulated during innate immunity by bacterial products, growth factors, cytokines, thrombin, and collagen. Most known eicosanoids from COXs were identified and structurally characterized in the 1980–1990s and include platelet-derived lipids, TXA_2_ and 12-HETE, as well as the PGs, exemplified by PGE_2_, and D_2_, well known as mediators of pain, fever, cell proliferation, and innate and adaptive immune responses. Our observation of a cellularly generated DX eicosanoid defines a new class of these lipids formed endogenously by mammalian cells. More members of this class are possible, given recent observations of purified LOXs being able to generate DX isomers via oxidation of epoxides *in vitro* in acellular experiments (14).

## Author Contributions

C. H., M. A., C. U., S. A., D. A. S., S. N. L., K. A. R., and C. P. T. conducted the experiments. C. H., P. W. C., M. A., V. O. D., R. C. M., and C. P. T. designed the experiments. L. J. M., H,. J. L., and T. D. W. provided reagents or patient samples. C. H. and V. O. D. wrote the paper. All authors edited the paper.

## Supplementary Material

Supplemental Data

## Footnotes

4 The abbreviations used are:

TXA_2_: thromboxane A_2_
DX: dioxolane
fMLP: formylmethionylleucylphenylalanine
DXA_3_: dioxolane A_3_
PAR: protease-activated receptor
cPLA_2_: cytosolic phospholipase A_2_
HETE: hydroxyeicosatetraenoic acid
PG: prostaglandin
NMBHA: *N-*methyl benzohydroxamic acid
ANOVA: analysis of variance
HPETE: hydroperoxyeicosatetraenoic acid
FTMS: Fourier MS
CID: collision-induced dissociation
AA: arachidonate
PLC: phospholipase C
LOX: lipoxygenase.
