# Supplementary material for: Human Platelets Utilize Cycloxygenase-1 to Generate Dioxolane A3, a Neutrophil-activating Eicosanoid
Source: J Biol Chem. 2016 Apr 22;291(26):13448–64. doi: 10.1074/jbc.M115.700609 (PMC4919433; doi:10.1074/jbc.M115.700609)
Supplement: Supplemental Data [file supp_291_26_13448__index.html]

Human platelets utilize cycloxygenase-1 to generate dioxolane A3, a neutrophil activating eicosanoid — Human Platelets Utilize Cycloxygenase-1 to Generate Dioxolane A3, a Neutrophil-activating Eicosanoid — A New Bioactive Eicosanoid Generated by Human Platelets — Supplemental Data 

# Human Platelets Utilize Cycloxygenase-1 to Generate Dioxolane A3, a Neutrophil-activating Eicosanoid

## Supplemental Data

- Supplementary Data (.pdf, 378 KB) - Supplementary Results and Figures
