# Supplementary material for: Human Platelets Utilize Cycloxygenase-1 to Generate Dioxolane A3, a Neutrophil-activating Eicosanoid
Source: J Biol Chem. 2016 Apr 22;291(26):13448–64. doi: 10.1074/jbc.M115.700609 (PMC4919433; doi:10.1074/jbc.M115.700609)

Supplementary Data.

Supplementary Results

Derivatization, acid hydrolysis and catalytic hydrogenation of DXA_3

DXA_3 generated by COX-1 oxidation of AA was partially purified using reverse phase HPLC, as described in Supplementary Methods, derivatized using PFB, MOX and/or TMS, and analyzed using GC/MS and/or LC/MS. First, LC/MS of MOX-derivatized DXA_3 showed no loss of lipid, indicating that it does not contain any carbonyl groups (Supplementary Figure 1 A,B). In contrast, a small amount of contaminating PGE_2 was lost on derivatization. It was not possible to analyze TMS-ethers using LC/MS/MS due to derivatization of the carboxyl group by the reagent (required for PFB derivatization and negative ionization and detection). Derivatization of DXA_3 using PFB, then MOX and TMS followed by GC/MS analysis, yielded signals at *m/z* 423 (+TMS), indicating one hydroxyl (Supplementary Figure 1 C). Note that during negative ion chemical ionization (NICI) analysis, the PFB group is lost generating the carboxylate anion, which is detected as a negative signal. Also, several lipids are detected on derivatization since three isomers as generated by COX-1 (Figure 7 C). Ions were not detected for addition of carbonyls or more than one hydroxyl (not shown). The MS spectrum at 11.4 min shows a molecular ion of *m/z* 423.2 and a fragment at *m/z* 333.1 (-90 amu), representing the loss of –OTMS (Supplementary Figure 1 D). This further supports the presence of a single hydroxyl group. Since the remaining two oxygens are neither hydroxyl or carbonyl, this is consistent with the proposed dioxolane structure. Epoxides, but not dioxolanes are acid sensitive. Thus, purified DXA_3 was incubated with 1% acetic acid, and analyzed using LC/MS. No loss of *m/z* 351 was observed (Supplementary

Figure 1 E,F). We note that DXA_3 will be considerably more lipophilic than PGE_2 or D_2, which both contain additional hydroxyl and carbonyl functional groups, consistent with our observation of later elution on reverse phase HPLC (Figure 1 A).

Supplementary Figure Legends.

Supplementary Figure 1. **Derivatization and analysis of DXA_3 using LC/MS/MS and GC/MS, shows the presence of one hydroxyl and no carbonyl or epoxide functional groups**. *Panels A,B. LC/MS shows DXA_3 contains no carbonyl groups.* Semi-purified DXA_3 generated using COX-1 was derivatized using methyloxime (MOX) and analyzed using LC/MS/MS, on the 2000 Q-Trap, monitoring *m/z* 351 in Q1. *Panel A*, no derivatization. *Panel B*, after derivatization. *Note*: loss of small peak corresponding to contaminating PGE_2 in this preparation confirms derivatization has been successful. *Panels C,D. GC/MS shows that DXA_3 contains one hydroxyl group and no carbonyls.* Purified DXA_3 generated using COX-1 was derivatized using PFB, MOX and TMS, and analyzed using GC/MS as described in Methods. *Panel C. m/z* of derivative with one hydroxyl, showing a major peak at 11.38 min, and two additional smaller peaks at either side. *Panel D.* MS spectrum at 11.38 shows ion at m/z 432, with loss of 90 amu at *m/z* 333 (-OTMS). *Panels E,F. DXA_3 is insensitive to acid hydrolysis indicating no epoxide groups.* Semi-purified DXA_3 generated by COX-1 was solubilized in acetonitrile before the addition of 1 % acetic acid (1:4). Following 30 min at 22 °C, lipids were extracted using a C_{18} Bond Elute cartridge and analyzed using LC/MS/MS on the 2000 Q-Trap for *m/z* 351.2 in full scan Q1 mode. *Panel E:* no hydrolysis, *Panel F*: after hydrolysis.

Supplementary Figure 2. Characterization of DXA_3-d8 MS/MS and MS^3 fragmentation, using high resolution FTMS. *Panel A. LC/MS/MS of COX-1 derived DXA_3-d8, generated using AA-d8 as substrate.* Analysis was undertaken on the Orbitrap Elite in FTMS mode, separating using reverse phase LC, isolating *m/z* 359.27 in the Velos Pro, then fragmenting using CID at 50 V, with resolution 15,000 ppm, as described in Methods. *Panel B. MS^3 of daughter ion at m/z 340.25 (smaller ion adjacent to m/z 341 in Panel a), with CID 30V. Panel C. MS^3 of daughter ion at m/z 322.24, with CID of daughter ion at 30V. Panel d. Proposed fragmentation pathway for m/z 359.2679 generating m/z 341.2573, which fragments to m/z 278.2507 via via m/z 322.2405.* DXA_3 loses H_2O forming m/z 341.2573. Following ring opening m/z 340.2511 , leaving a keto group at C9, H_2O and CO_2 are lost, generating m/z 278.2507via a m/z 322.2405 intermediate, as shown.

Supplementary Figure 3. Characterization of DXA_3-d8 MS/MS and MS^3 fragmentation, using high resolution FTMS. *Panel A. MS^3 of daughter ion at m/z 231.15 with CID 30V. Panel B. MS^3 of daughter ion at m/z 212.13, with CID 30V. Panel c. Proposed fragmentation pathway for m/z 359.2 generating m/z 358.2, then via fragmentation of 340.2, forming 212.1 and 168.1.* Following ring opening, with keto group at C11, H_2O is lost, followed by two 1[5]-sigmatropic shifts generating *m/z* 340.2511. Following loss of a conjugated triene, *m/z* 231.1509 is generated, which then loses H_2O, and *via* an intermediate fragments to *m/z* 212.1341 and last 168.1422.

Supplementary Figure 1

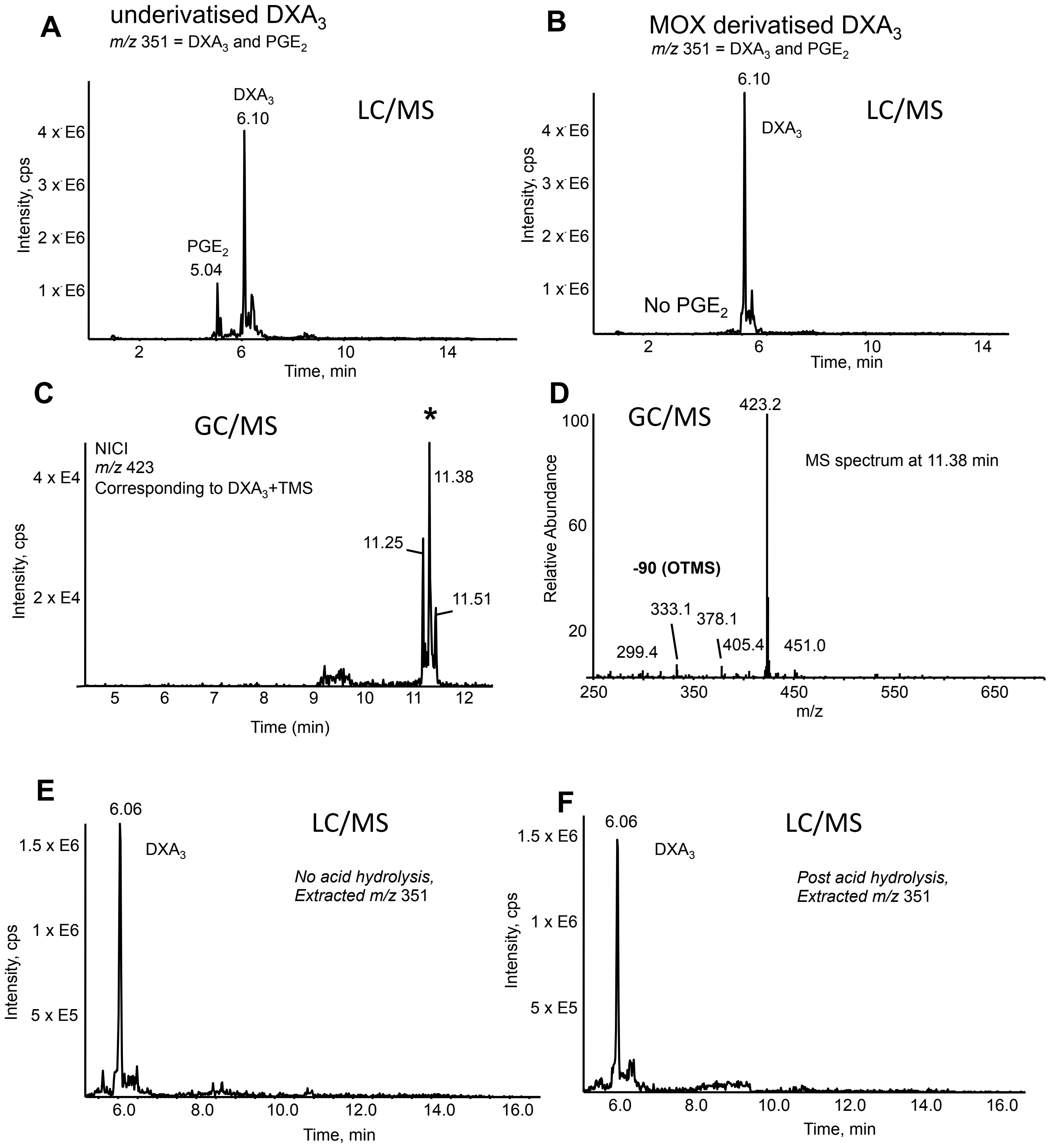

Supplementary Figure 2

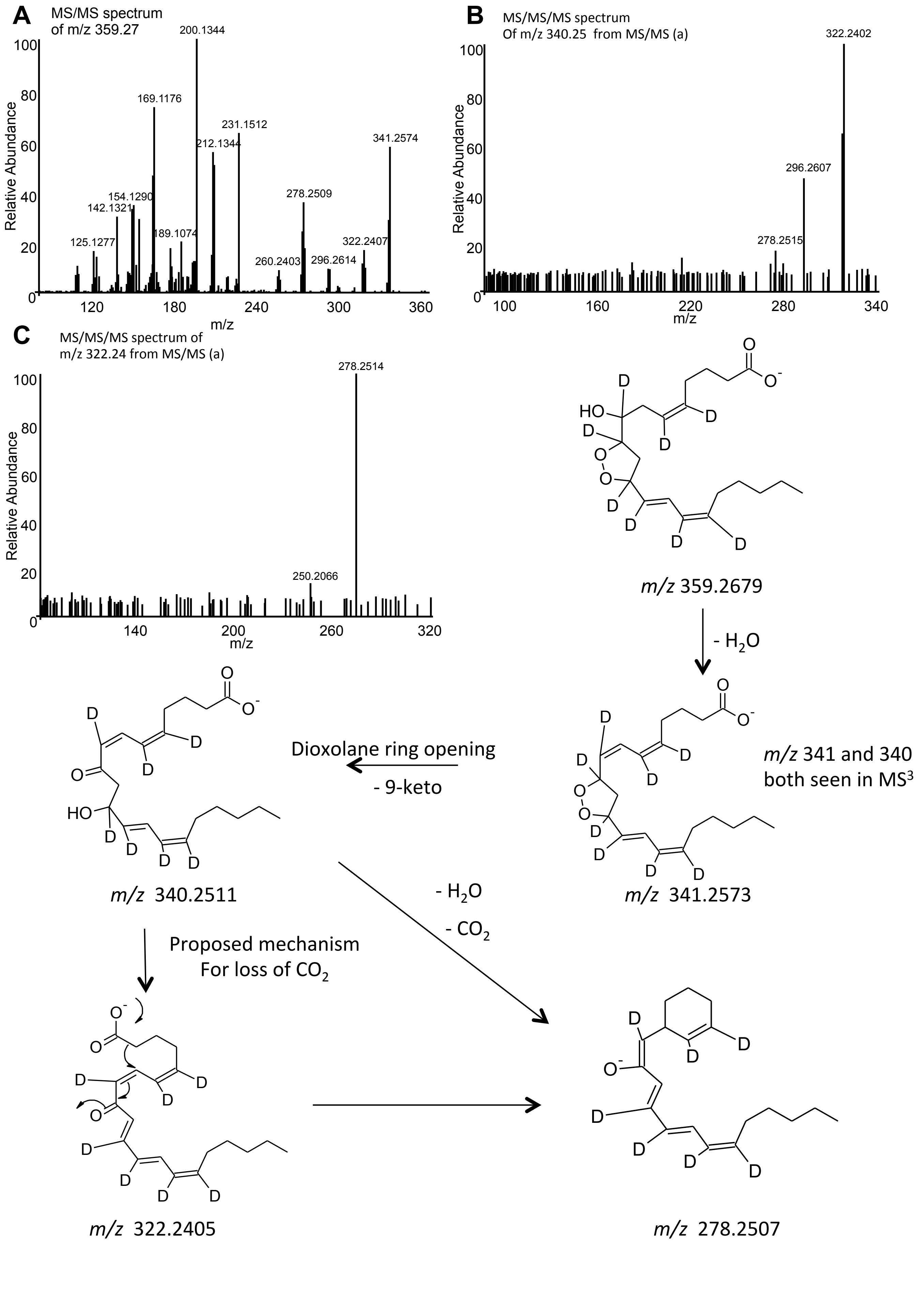

Supplementary Figure 3

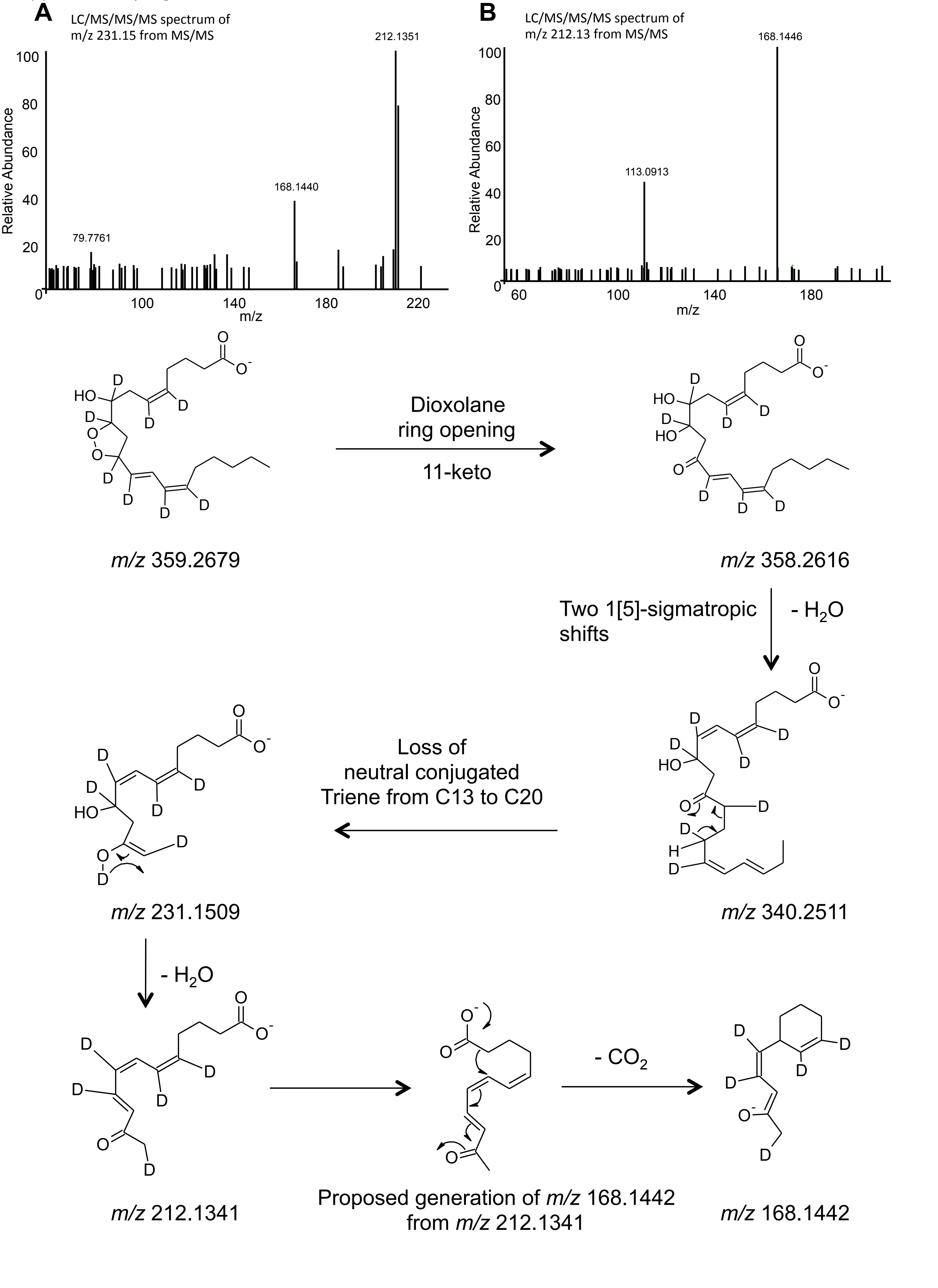

Supplement: Supplemental Data [file 10.1074_M115.700609_jbc.M115.700609-1.pdf]
